# Facile Process for Surface Passivation Using (NH_4_)_2_S for the InP MOS Capacitor with ALD Al_2_O_3_

**DOI:** 10.3390/ma12233917

**Published:** 2019-11-27

**Authors:** Jung Sub Lee, Tae Young Ahn, Daewon Kim

**Affiliations:** 1 Department of Orthopaedic Surgery and Medical Research Institute, Pusan National University Hospital, 179 Gudeok-ro, Seo-gu, Busan 49241, Korea; 2 Department of Electronic Engineering, Institute for Wearable Convergence Electronics, Kyung Hee University, 1732 Deogyeong-daero, Giheung-gu, Yongin 17104, Korea

**Keywords:** III–V semiconductor, indium phosphide (InP), Al_2_O_3_, (NH_4_)_2_S, sulfur passivation

## Abstract

Ammonium sulfide ((NH_4_)_2_S) was used for the passivation of an InP (100) substrate and its conditions were optimized. The capacitance–voltage (C–V) characteristics of InP metal-oxide-semiconductor (MOS) capacitors were analyzed by changing the concentration of and treatment time with (NH_4_)_2_S. It was found that a 10% (NH_4_)_2_S treatment for 10 min exhibits the best electrical properties in terms of hysteresis and frequency dispersions in the depletion or accumulation mode. After the InP substrate was passivated by the optimized (NH_4_)_2_S, the results of x-ray photoelectron spectroscopy (XPS) and the extracted interface trap density (D_it_) proved that the growth of native oxide was suppressed.

## 1. Introduction

As silicon-based, complementary metal-oxide-semiconductor (CMOS) technology has been approaching its fundamental limits, III–V semiconductors have been focused on as alternative channel materials due to their high electron mobility. In particular, indium phosphide (InP) is considered one of the promising materials due to its larger band gap than silicon (~1.34 eV), which results in low off-state leakage current and high breakdown field [1,2,3]. In addition, InP is preferred to a barrier layer between the gate oxide and the In_x_Ga_1−x_As channel [4]. Despite these advantages, the poor interfacial quality between a high-k dielectric and an InP substrate compared to SiO_2_ and Si is still a major obstacle to overcome before high-performance MOS field-effect-transistors (MOSFETs) can be realized. Thus, every endeavor to keep the InP substrate clear of the native oxide growth and external contamination prior to deposition of the high-K dielectric is required for a reduction of D_it_. For this purpose, ex situ cleaning methods using wet chemicals, such as HCl, H_2_SO_4_, NH_4_OH, and HF, have consistently been developed [5]. Various works have been done for the enhancement of the interfacial quality over the last few decades [6,7,8]. Recently, Cuypers et al. reported that sulfur passivation by (NH_4_)_2_S was useful for suppressing unwanted effects arising from pre-existing defects and the rapid re-oxidation of the surface after wet chemical cleaning [9]. As the group VI element, sulfur and oxygen have the same number of valence electrons; however, the electronegativity of sulfur is lower than that of oxygen. Hence, the interface of In–S become stable so that the formation of the native oxide can be effectively inhibited. To take advantage of this feature, (NH_4_)_2_S has widely been employed [1,2,3,10,11,12,13]. However, comprehensive analyses regarding the process parameters used in sulfur passivation which affect the InP MOSFETs remain insufficient.

In this work, the conditions of (NH_4_)_2_S passivation were optimized to enhance the performance of the device. The electrical characteristics of an InP MOS capacitor (MOSCAP) were investigated while varying the passivation conditions of (NH_4_)_2_S, in this case the concentration and treatment time. Then, the chemical properties of the sulfur-passivated interface with the optimized conditions were compared with those of a non-passivated interface. Furthermore, the D_it_ distribution across the InP energy bandgap was extracted using a conductance method [14,15,16,17] in order to evaluate the interfacial quality, which affects the performances of InP MOSFETs.

## 2. Experimental

MOSCAPs were fabricated on an undoped InP (100) wafer with an n-type carrier concentration of 5 × 10^15^ cm^−3^. Initially, the substrates were cleaned with a 1% HF solution for 5 min, after which they were treated with (NH_4_)_2_S at concentrations of 1%, 5%, 10%, and 22% which were diluted by deionized H_2_O for 10 min at room temperature (300 K). Other substrates were treated with the fixed concentration of 10% (NH_4_)_2_S for 5 min, 10 min, and 30 min. As the gate oxide, 7 nm of Al_2_O_3_ was deposited on the substrate at a temperature of 150 °C by means of atomic layer deposition (ALD) after a rinse by water was performed. In the ALD process, trimethylaluminum (TMA) and H_2_O were sequentially supplied under purging with N_2_ during each deposition cycle. For a gate electrode, 10 nm of Ni and 100 nm of Au were deposited using a thermal evaporator through a shadow mask. Afterwards, the MOSCAPs were annealed with ambient gas (4% H_2_/96% N_2_) at 400 °C for 30 min.

## 3. Discussion and Results

The structural properties of the Au/Ni/Al_2_O_3_/InP samples were characterized by high-resolution transmission electron microscopy (HR-TEM) using a field-emission transmission microscope operated at 300 kV (Tecnai G2 F30). In order to study the bonding status at the Al_2_O_3_/InP interface, ex situ X-ray photoelectron spectroscopy (XPS) was analyzed using a monochromated Al Κα (1486.7 eV) source with a base pressure in the mid-10^−10^ Torr range. Capacitance-voltage (C–V) and conductance–voltage (G–V) measurements were carried out using an E4980A precision LCR meter at room temperature.

**Figure 1:**
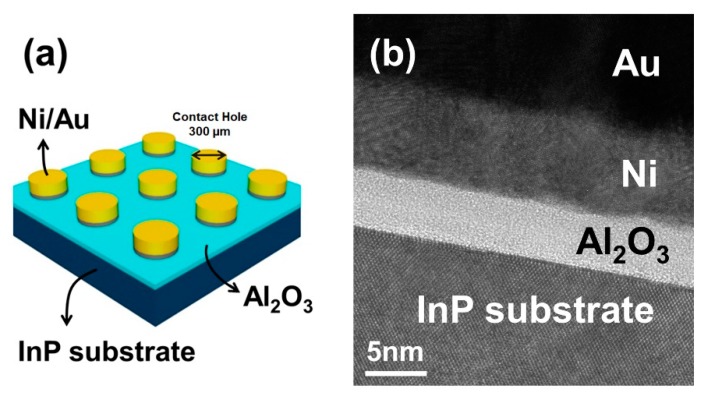
(**a**) Device schematic for the InP metal-oxide-semiconductor (MOS) capacitor. (**b**) HR-TEM image of 7 nm thick Al_2_O_3_ on the InP (100) substrate.

A schematic of the MOSCAP is shown in Figure 1a. Al_2_O_3_ was deposited over the entire InP substrate. Afterwards, patterned Ni and Au were deposited through a shadow mask which had holes with a diameter of 300 μm. Figure 1b shows a cross-sectional HR-TEM image of the MOSCAP on the InP substrate, which was treated with the 10% (NH_4_)_2_S solution for 10 min. The TEM image indicates that the ALD Al_2_O_3_ has a uniform thickness and an amorphous structure. Moreover, an abrupt interface was created without the growth of the native oxide.

**Figure 2:**
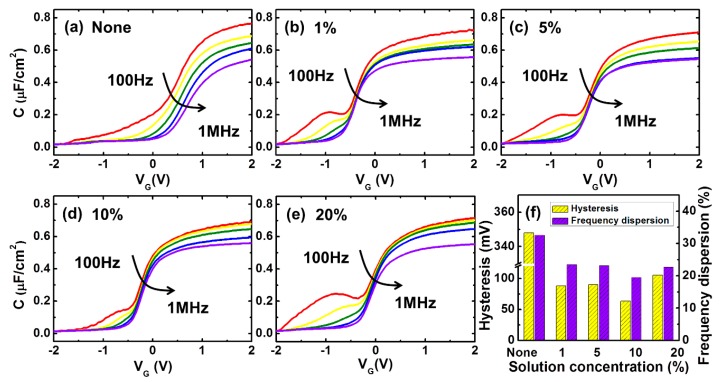
The capacitance–voltage (C–V) characteristics of (**a**) 0%, (**b**) 1%, (**c**) 5%, (**d**) 10%, and (**e**) 22% (NH_4_)_2_S treated Au/Ni/Al_2_O_3_/InP. They were measured at room temperature under various frequencies of 100 Hz, 1 kHz, 10 kHz, 100 kHz, and 1 MHz. (**f**) Measured hysteresis at 100 kHz for all samples. Frequency dispersion of the accumulation capacitance ranging from 100 Hz to 1 MHz (four decades).

Figure 2a–e shows the C–V characteristics of the devices treated with diluted (NH_4_)_2_S solutions at different concentrations. Three issues exist in all of the curves. First, the accumulation capacitance cannot reach the theoretical value of the oxide capacitance, which is calculated according to the actual thickness of the gate dielectric. This phenomenon originates from the low density of states (DOS) of the InP substrate above the conduction band. Secondly, there is frequency dispersion in the depletion mode, an indicator of the level of D_it_, which is an important characteristic in terms of enhancing the performance of MOSFETs. Lastly, there is also frequency dispersion in the accumulation mode. This is associated with conductive losses, which are mostly caused by border traps located in the gate dielectric near the interface [16,18,19]. As shown in Figure 2f, hysteresis and frequency dispersion in the accumulation are effectively suppressed in the sample treated with the 10% (NH_4_)_2_S solution. Consequentially, the smallest D_it_ and border trap distribution were achieved by the 10% (NH_4_)_2_S treatment, whereas (NH_4_)_2_S concentrations of 1% and 5% were not sufficient for substrate passivation, and a concentration of 22% resulted in excessive surface roughness [20].

Subsequently, based on a concentration of 10%, a short-term treatment time such as 5 or 10 min is proper for passivation, while a longer treatment time such as 30 min could attack the surface akin to the process with the 22% solution at 10 min (refer to the Supplementary Figure S1). On the other hand, other concentrations are also able to passivate the substrate, but they are not appropriate, because the 1% and 5% solutions require longer treatment times and 22% causes excessive surface roughening; there are no additional advantages compared with 10%. In this regard, another substrate, such as In_0.53_Ga_0.47_As [8,21] or InSb [20], shows the same trend depending on the concentration and treatment time of the solution. Therefore, the 10% (NH_4_)_2_S solution for 10 min is an optimal passivation condition for the InP (100) as well as other III–V substrates to attain the best electrical characteristics.

Using the 10% (NH_4_)_2_S treatment as the optimum condition for InP substrates, the interface characteristics of Al_2_O_3_/InP were further studied through an analysis of the chemical properties and D_it_ distributions beyond the electrical properties.

**Figure 3:**
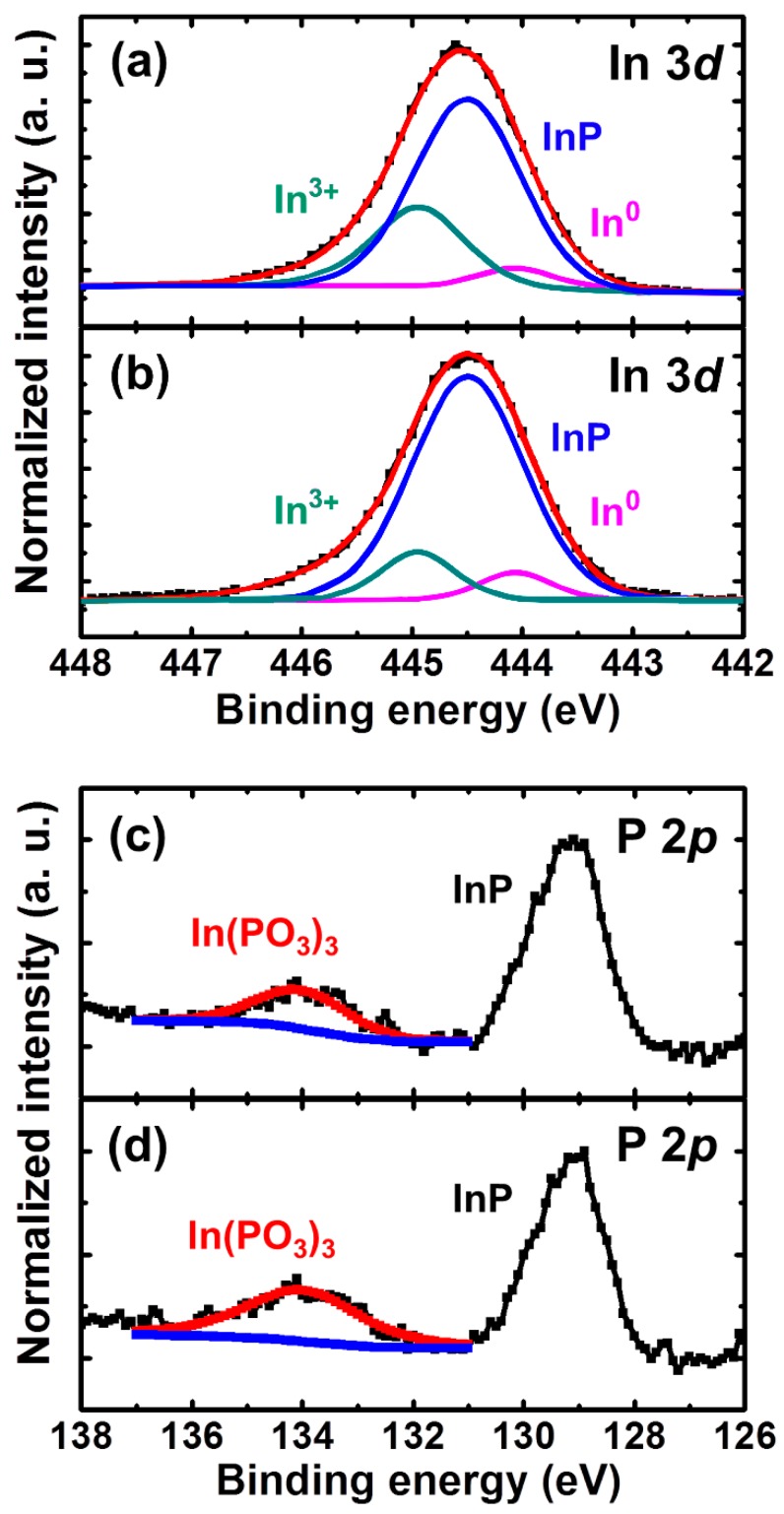
The In 3*d* core level XPS spectra of the sample (**a**) with or (**b**) without 10% (NH_4_)_2_S treatment. The P 2*p* core level spectra of the sample (**c**) with or (**d**) without 10% (NH_4_)_2_S treatment.

Figure 3a,b shows the In *3d* core level spectra of InP substrates with or without sulfur passivation, respectively. There are three deconvoluted peaks caused by the native oxides of indium oxides—InO, InPO_4_/In(PO_3_)_3_, and InPO_x_ with binding energy levels of 444.9 eV, 445.5 eV, and 446.1 eV, apart from another peak caused by the InP substrate with a binding energy of 444.4eV [9,22]. Importantly, according to a comparison of Figure 3a,b, the peaks related to InPO_4_/In(PO_3_)_3_ and InPO_x_ were reduced due to sulfur passivation, which prevented the native oxide from growing. A similar trend was also observed at P 2*p* core level spectra of the InP substrate, as shown in Figure 3c,d. There were two peaks caused by In(PO_3_)_3_ and the InP substrate with binding energies of 134.1 and 129 eV [9,22]. In addition to the In 3*d* spectra, the P 2*p* spectra also indicated that the native oxide was reduced due to sulfur.

**Figure 4:**
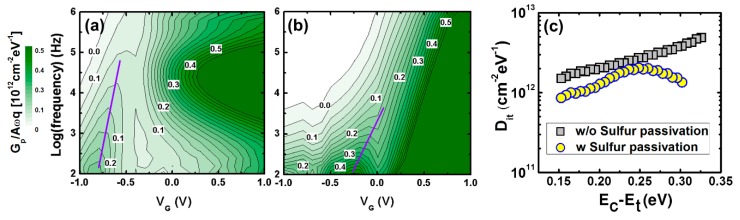
Contour map of normalized conductance (G_p_/ω)/Aq as a function of frequency and gate bias for MOSCAPs (**a**) without or (**b**) with 10% (NH_4_)_2_S treatment. (**c**) D_it_ distribution across the InP energy bandgap extracted from the conductance method for MOSCAPs with or without 10% (NH_4_)_2_S treatment.

Figure 4 illustrates G–V contour maps and the D_it_ extracted from the InP MOSCAPs with or without sulfur passivation. These contour maps exhibit the magnitude of the normalized parallel conductance, (G_p_/ω)/Aq, versus both the AC frequency and the gate voltage, where ω is the frequency, A is the area of the MOSCAP, and q is the elementary charge. From the contour maps, the line to connect the maximum value of (G_p_/ω)/Aq represents the band bending efficiency and the Fermi level movement with gate bias [15,23,24]. The maximum value of (G_p_/ω)/Aq increases with a steeper slope with the 10% (NH_4_)_2_S compared to the case with no sulfur passivation, as shown in Figure 4a,b. Therefore, it should be noted that the sulfur passivation leads to the depinning of the Fermi level, making the band bending more efficient. Moreover, border traps were validated again. In the case with no passivation, the (G_p_/ω)/Aq peak spreads over the entire frequency range in the accumulation, compared to the case with 10% (NH_4_)_2_S passivation. This implies that the conductive loss is caused by two factors: border traps and series resistance. The maximum value of (Gp/ω)/Aq extracted by the measurement equipment was used as the level of the Dit distribution across the maps. Figure 4c indicates that the MOSCAP with a sulfur treatment shows a lower D_it_ distribution than that without a sulfur treatment. This result is consistent with the C–V characteristics shown in Figure 2a,d.

## 4. Conclusions

The effects of (NH_4_)_2_S passivation with various concentrations and treatment times were investigated. Two characterization methods, an electrical method and a chemical method, were employed for comprehensive analyses of the surface properties. In terms of the electrical characteristics, the 10% (NH_4_)_2_S treatment for 10 min was the optimal condition to improve the electrical properties of devices built on InP (100) substrates. This was supported by both the C–V plot and the G–V contour map in terms of the frequency dispersions and hysteresis. In terms of the chemical characteristics, the device with the 10% (NH_4_)_2_S treatment for 10 min showed relatively low native oxide peaks, particularly arising from the phosphorus-related oxide compared to that without sulfur passivation. This was supported by XPS data. Finally, it was also found that the level of D_it_ was lower in the device with the 10% (NH_4_)_2_S treatment than in the device with no sulfur passivation. The proposed strategy to optimize the conditions of diluted (NH_4_)_2_S provides a guideline for further improvements of InP-based high-performance CMOS technology.

## Supplementary Materials

The following are available online at https://www.mdpi.com/1996-1944/12/23/3917/s1, Figure S1: The capacitance-voltage characteristics of the Au/Ni/Al_2_O_3_/InP which was treated on 10% (NH_4_)_2_S for (a) 5, and (b) 10, and (c) 30 min. Measurement frequencies are 100 Hz, 1 kHz, 10 kHz, 100 kHz, and 1 MHz.
