# Supplementary material for: Facile Process for Surface Passivation Using (NH4)2S for the InP MOS Capacitor with ALD Al2O3
Source: Materials (Basel). 2019 Nov 27;12(23):3917. doi: 10.3390/ma12233917 (PMC6926807; doi:10.3390/ma12233917)
Supplement: Supplementary file 1 [file materials-12-03917-s001.pdf]

materials

MDPI

Supplementary Information

Facile Process for Surface Passivation Using $(NH_4)_2S$ for the InP MOS Capacitor with ALD Al_2O_3

Jung Sub Lee [1], Tae Young Ahn [1,*] and Daewon Kim [2,*]

[1] Department of Orthopaedic Surgery and Medical Research Institute, Pusan National University Hospital, 179 Gudeok-ro, Seo-gu, Busan 49241, Korea; jungsublee@pusan.ac.kr

[2] Department of Electronic Engineering, Institute for Wearable Convergence Electronics, Kyung Hee University, 1732 Deogyeong-daero, Giheung-gu, Yongin 17104, Korea

* Correspondence: daewon@khu.ac.kr (D.K.); falconbleu@daum.net (T.Y.A.)

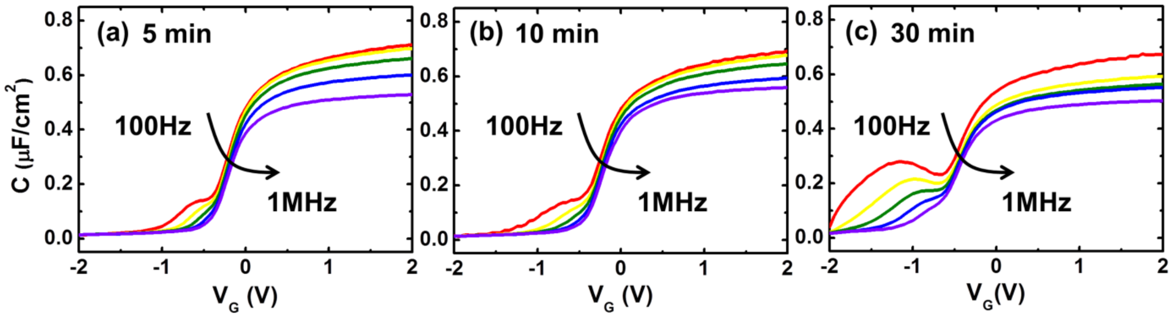

Figure S1. The capacitance-voltage characteristics of the Au/Ni/Al_2O_3/InP which was treated on 10% $(NH_4)_2S$ for (**a**) 5, and (**b**) 10, and (**c**) 30 min. Measurement frequencies are 100 Hz, 1 kHz, 10 kHz, 100 kHz, and 1 MHz.

© 2019 by the authors. Licensee MDPI, Basel, Switzerland. This article is an open access article distributed under the terms and conditions of the Creative Commons Attribution (CC BY) license (http://creativecommons.org/licenses/by/4.0/).
